# The Evolution of the Modern Hospital

**Published:** 1915-02-20

**Authors:** B. Burford Rawlings


### THE EVOLUTION OF THE MODERN HOSPITAL.

### I.—The Internal Revolution.

By B. BURFORD RAWLINGS.

The past forty years have witnessed many great social changes, and nowhere have those changes been more complete, or more indicative of the spirit of the age, than in respect of the provision made for the medical treatment of the sick poor.

In the case of the hospitals it has not been reform, but revolution. Alike in their habitations, their government, and all the details of their domestic life, the old order of things has passed away, and we have witnessed the dawn of a new era. It has come to be recognised that what is provided must be of the best, that the buildings of our hospitals, their sanitation, equipment, and general accommodation must reflect the advanced knowledge of the day, and that the science of the physician and of the surgeon can be turned to the best account only when the conditions for its exercise are favourable, and when it is seconded by a generous administration and efficient nursing. Beyond this it is also understood that help without sympathy is a mean and sordid gift, and that our duty to the sick is not limited to a supply of their bare necessities, but comprehends an ungrudging endeavour to surround the patient with all that can conduce to his cheerfulness and comfort and the discharge of those numberless little offices which tell of tender and unwearying solicitude for his welfare.

To those familiar only with the hospitals as they are a glimpse of their condition forty years ago would be startling. Intelligent interest in the hospitals has grown, and if even now there is not enough, forty years ago there was none. The actual workers, doubtless, showed concern for their particular institutions, and were not wanting in industry and devotion, but for the most part the hospitals seem to have been abodes of sleepy inactivity. Their managers were content to pursue the course marked out by their predecessors, and, being comfortable in their ignorance of defects, were undisturbed by desire for improvement.

It might be argued, not unreasonably, that in the early Victorian period many of the sick and injured who depended upon charitable relief were in worse case, apart from the services of the physicians and surgeons, than their ancestors of mediæval times. The lodging and tendance provided for them were inferior to the shelter of the religious houses and the ministrations of Brotherhoods inspired by an enthusiasm of philanthropy and obedient to the exactions of an exalted sense of duty and religion.

The hospitals themselves, as buildings, had much in common with the prisons. In most cases they were gloomy and grimy places, bereft externally of architectural features, whose heavy portals opened into bare, sun-forsaken passages leading to cavernous wards unrelieved by a particle of colour and unequipped with a single concession to comfort, while sanitation as an applied science can scarcely be said to have existed. These wards depended for daylight upon windows whose least and last office would appear to have been its admission. In the winter semi-darkness prevailed at noon, and what artificial illumination was provided proceeded from naked gas jets usually hung high in the centre of the ward, always inclined to flare, and effective chiefly in blackening the ceiling and casting dancing shadows upon beds borne down with the weight of restless sufferers. When a close inspection of a patient was necessary the grizzled nurse held above her head a candle charged with a portentous wick, which we may be sure she habitually snuffed with her fingers.

The severity and unsuitability of the building and fittings extended to the whole establishment, and it is easy to believe that the patients, sensitive to their environments, as sick folk usually are, must have been caused much unnecessary distress when removed from the familiar surroundings of even the humblest home to the forbidding desolation of a vast, ill-ventilated, and unsweetened chamber, containing disease in every stage, and where little or no attempt was made to cloak the nakedness of horrors inseparable from any aggregation of human suffering.

And if the material arrangements were repulsive, still more unsatisfactory, because they were still more important, were those for nursing and treatment. The world has been recently reminded of the ordeals of sufferers before anæsthetics were brought to the aid of surgery. Scarcely less real were the horrors preliminary to surgical operation when all the preparations were made in view of the patient, and, unless unconsciousness had mercifully intervened, everything which could add to his terror was brought to his notice. To suggest that the spirit of humanity was wanting would not be reasonable. We may be sure it shone brightly in individuals, and if the unwritten records of the hospital life of those days could be searched they would reveal many an instance of heroism and self-sacrifice. But the circumstances were not favourable to the virtues which, if decorum, or even decency, is to be preserved, must be displayed by all to whose charge the sick and helpless are committed. There was no intercommunication between the outer world and the inner life of our hospitals, and consequently no high standard of hospital ethics. Organised lay visitation of hospital wards was unknown. They were the days when the oft-quoted Bob Sawyer and Mrs. Gamp really were types of their classes; not the grotesque caricatures they now appear, and when not even the visiting members of the staff could be described as conspicuous, commonly, for refinement and good breeding. Those, too, were the days when the whole internal economy of the hospital was something to be wondered at; when there was no range of dietary for the patients such as is now considered indispensable to the well-being of sick people, no adequate supply of nurses, and no nursing in the sense we now understand the phrase, because there was no training. Neither was there any suitable accommodation for the women called nurses, who in knowledge and station were little better than charwomen, and in some hospitals were actually interchangeable with the scrubbers. No separate apartments were provided for them; they were lodged wherever space could be found, taking their meals in the kitchen with the servants, sleeping sometimes in big bare dormitories, sometimes in cubicles in the wards, sometimes in out-of-the-way corners of corridors, in basement rooms little better than cellars, in one case under the stairs, and rarely, if ever, in privacy and comfort.

Here, as often happens, cause was confounded with effect. While no effort was made to induce respectable women to come forward, hospital authorities were content to believe that none but the dregs of womanhood would undertake duties so repulsive, a view which appeared to be corroborated by facts, as we read that when a call was made in 1847 for nurses to take up work in Haslar Hospital, not one person responded.

Yet the influence of outside opinion, uninstructed and feeble as it must have been, was not wholly absent, and its value received a remarkable illustration in the fact that while the condition of the voluntary hospitals was bad, that of the Poor-Law infirmaries was infinitely worse. No light from the outside reached them, and in the abominations of their internal condition they reflected only too accurately the government of Bumbledom. The voluntary hospitals were at least regarded as "Charities": they were the outcome of the munificence or the labours of pious founders, and they had some traditions to maintain and some reputation to guard. The governing bodies were composed of gentlemen, not very active, it is to be feared, and not very capable or far-seeing, but at least their sense of duty was not exhausted in an effort to keep down the rates, and they were not without sympathy for suffering. They were, however, fettered by custom and shut in by the prejudices grown up about them. They had no outlook beyond the conventional; they possessed no freedom, and just as the majority of men engaged in hospital management at the present day often, in spite of their convictions, shrink from the thought of assailing the dogma of medical infallibility, so those good people would have repelled with alarm and horror a proposal for any of the reforms which, happily for the sick and the nation, have been brought about by the labours of a few zealots endowed with unflinching energies and an intelligence almost inspired.

Our hospitals demanded attention and reform both in respect of their accommodation and their *personnel.* The relationship between the two was close, and it has been abundantly shown that, given improvement in the quality of the worker, other advances follow consequentially. The hospital first to be erected in conformity with the notions and requirements of latter-day science was the new St. Thomas's. The old building, situated in proximity to Guy's, was a fair example of the architectural style which satisfied the aspirations of the seventeenth century, and it might have stood to the present day had not an exercise of the arbitrary powers accorded by Parliament to a railway company compelled the governors to remove to another site.

But although it was the action of the company which brought matters to a crisis, other causes predisposing to the substitution of a new building for the obsolete and inconvenient fabric of the old St. Thomas's had not been wanting. To the intelligent observer disquieting signs had appeared. It was suspected that the art and theory of nursing, that portion of the general work of attending the sick which in importance is secondary only to the ministrations of the physician, and not always even secondary, was woefully misunderstood, and that in practice it was defective in many important particulars.

